# Juvenile polymyositis and leptospirosis association: case report

**DOI:** 10.1186/1546-0096-12-S1-P280

**Published:** 2014-09-17

**Authors:** Luciana B Paim-Marques, Morganna Andrade, Lincoln Mesquita

**Affiliations:** 1 Pediatric Rheumathology, Infantil Albert Sabin Hospital, Fortaleza, Brazil; 2 Pediatric rheumathology, Fortaleza University, Fortaleza, Brazil

## Introduction

The Polymyositis (PM) is an inflammatory myopathy with symmetrical proximal muscle weakness, especially in pelvic, shoulder girdle and cervical musculature. It may occur primary or secondary to other diseases. Diagnosis is made by exclusion of other etiologies, such as infections, endocrinopathies, metabolics diseases and Bohan and Peter criteria.

Leptospirosis is a zoonosis of worldwide distribution, that can be asymptomatic, with clinical presentation ranging from an acute febrile illness, with headache, severe myalgia, fever, arthralgia, to a severe syndrome of multiorgan dysfunction (Weill syndrome) and the diagnosis may be missed unless the physician has a high index of suspicion for the disease.

## Objectives

Describe an adolescent with juvenile polimyositis caused by a leptospirosis infection.

## Methods

Patient 14 years, female , reports that two weeks before admission , she developed fever, diffuse rash , arthritis of the left ankle with progressive ascendant loss of force in lower and upper limbs associated with progressive dysphagia initially to solids and later to liquids. The patient had absent patellar reflexes, decreased proximal muscle strength (grade II), CHAQ: 6,8. Initial laboratorials tests : creatine kinase (CPK): 8438 ; Aldolase : 110 U/L; AST : 236U/L ; ALT : 141U/L, lactic dehydrogenase (LDH): 2468 U/L, cervical MR: normal, serologies: HIV, Epstein Barr , Hepatitis A, B and C, coxsackie B, Herpes, cytomegalovirus , parvovirus and dengue were negative, but leptospirosis serology was IgM positive and IgG negative. Patient received crystalline penicillin for 10 days , as recommended , but did not improved muscle strength and enzymes . Electroneuromyography showed myopathic pattern and subsequent muscle biopsy: inflammatory myositis; ANA, anti-μi-2 and anti-Jo-1 negatives. Patient improved after methylprednisolone pulses, and subsequent treatment with oral prednisone , associated with methotrexate.

## Results

There are reports of infectious diseases preceding inflammatory muscle diseases which may suggest an association between late infections and inflammatory diseases . The association of PM with leptospirosis has been described in few cases in the literature . Leptospirosis can occur in children mimicking a polymyositis and increasing CPK levels. There is a immunologic relationship of leptospira infection with atypical onset of inflammatory response , such as rheumatic manifestations. In our case , after the initial diagnosis , proper treatment and repeat serology (Ig M became negative), the patient did not improved and was subsequently diagnosed as juvenile inflammatory polymyositis.

## Conclusion

Leptospirosis is a condition that leads to clinical and laboratory muscular disorders and should be considered in endemic areas , it can simulate or trigger a polymyositis.

## Disclosure of interest

L Paim-Marques Paid Instructor for: novartis, Shire, M Andrade: None declared, L Mesquita: None declared.

